# Novel Nut and Bolt Task Quantifies Motor Deficits in Premanifest and Manifest Huntington’s Disease

**DOI:** 10.1371/currents.hd.ded251617ae62a1364506b0521bd3761

**Published:** 2015-09-08

**Authors:** Lucy M. Collins, Faye Begeti, Francesca Panin, Alpar S. Lazar, Travis Cruickshank, Mel Ziman, Sarah L. Mason, Roger A. Barker

**Affiliations:** John van Geest Centre for Brain Repair, Cambridge, United Kingdom; Faculty of Medical Sciences, Anglia Ruskin University, Cambridge, United Kingdom; John van Geest Centre for Brain Repair, Cambridge, United Kingdom; School of Medical Sciences, Edith Cowan University, Perth, Western Australia, Australia; School of Medical Sciences, Edith Cowan University, Perth, Western Australia, Australia; John van Geest Centre for Brain Repair, Cambridge, United Kingdom; John van Geest Centre for Brain Repair, Cambridge, United Kingdom; School of Medical Sciences, Edith Cowan University, Perth, Western Australia, Australia

## Abstract

Background: We investigated the use of a simple novel nut and bolt task in premanifest and manifest Huntington’s disease (HD) patients to detect and quantify motor impairments at all stages of the disease.

Methods: Premanifest HD (n=24), manifest HD (n=27) and control (n=32) participants were asked to screw a nut onto a bolt in one direction, using three different sized bolts with their left and right hand in turn.

Results: We identified some impairments at all stages of HD and in the premanifest individuals, deficits in the non-dominant hand correlated with disease burden scores.

Conclusion: This simple, cheap motor task was able to detect motor impairments in both premanifest and manifest HD and as such might be a useful quantifiable measure of motor function for use in clinical studies.

## INTRODUCTION

Huntington’s disease (HD) is an autosomal dominant condition causing neurodegeneration of the cortex^1^ white matter^2^^,^ 3 and basal ganglia^4^^,^ 5. This leads clinically to motor deficits including chorea, bradykinesia, and dystonia as well as cognitive deficits, circadian rhythm disturbances and psychiatric problems^6^^,^ 7. The ability to robustly detect early motor impairments in HD using a simple cheap test is needed to provide an objective quantifiable measure of motor performance that is useful both clinically and as an endpoint in therapeutic trials, and which can be followed over time. Also more tests that measure everyday motor tasks that impact on functional independence as the disease progresses are required. As such, many groups, including our own, have sought to develop motor tasks to detect and track early changes in premanifest HD (preHD)^8^^,^ 9 including both the finger^8^^,^ 9 and hand tapping tests 10 ^,^ 11 ^,^ 12 ^,^ 13. Motor differences are not just seen in tapping tasks, as other studies have shown that there are changes in the variability of grip force in a grasping task in HD patients 14; a reduction in tongue force in preHD patients 15; a reduction of speech rate in motor speech tasks 16 and impairments in saccade latency and velocity in HD 12 ^,^ 17. Furthermore these simple tasks have been correlated to structural brain changes in HD and preHD 18 as well as cognitive deficits 19. Other more complex motor tasks such as those involving diadochokinetic movements 20 and peg insertions, whilst able to detect impairments in manifest disease are insensitive during preHD 21. Many of these tasks described require specialized equipment and complicated analysis. In this paper we have assessed participants with manifest HD and preHD using a very simple and cheap nut and bolt test which has previously been used to look at the effects of extravehicular activity (EVA) gloves on dexterity, grip force and coordination in healthy participants 22. This new test is ideal for use in HD given its ecological validity, its ease of administration and the fact that it is so inexpensive; this makes it accessible for use in any population of HD patients and it doesn’t require any specialised custom computer software to interpret the results collected. To examine the validity of this new motor task, we tested a large group of individuals at pre-manifest and manifest stages of HD compared to healthy controls. Finally, we examined whether performance on this timed nut and bolt test was related to disease burden score (DBS). We now report for the first time that this simple inexpensive test can detect the earliest motor abnormalities in HD and performance correlates with disease burden scores across the entire disease, and so could be used to target those patients on the cusp of developing overt disease and as such may be suitable for use in new trials of disease modifying therapy.

## METHODS

**Participants**

Participants were recruited from the regional NHS HD clinic at the John Van Geest Centre for Brain Repair between 2012 and 2014. Control subjects with no known neurological disease were recruited from friends or relatives accompanying patients to the clinic. Written informed consent was taken and the nut and bolt data was collected under ethical approval [Reference number:09/H0308/2] approved by Cambridgeshire 2 Research Ethics Committee. To collect additional data the task was also expanded to include patients seen in clinic as part of their routine clinical assessment and the collection of this data was classified as a service evaluation with the Patient Safety Unit at Addenbrooke’s Hospital (Project Register Number: 4256). The work was carried out in accordance with the World Medical Association Declaration of Helsinki 23. All preHD and HD participants had a positive genetic test for the HD mutation and were assessed using the UHDRS motor examination along with their total functional capacity (TFC) sub-section 24. PreHD participants were defined by having a diagnostic confidence level on the UHDRS of less than 4. Disease burden score was calculated using the CAG-Age Product Scale (CAPS) 25 ^,^ 26.

**Nut and bolt task**

The nut and bolt task was designed as a simple test to examine dexterity of the hands and fingers. The participants were asked to screw a nut onto a bolt in one direction using either the left or right hand; participants were not allowed to flick the nut and the hand holding the bolt had to remain static/stationary. The task consisted of 3 different sized nuts and bolts and was timed for both hands for all conditions. The task was performed using two test conditions; while the hands were supported resting on a table or unsupported, with the hands held in mid air. The task was timed using a standard stopwatch, the timer was started when the participant began screwing the nut onto the bolt and the timer was stopped when the nut reached the top of the bolt and could go no further. All those administering this test were instructed in how to carry out the test, but the simplicity of the task and the use of a simple stopwatch meant that no extensive training was needed.

**Statistics**

Statistical analysis was performed using IBM SPSS software version 21.0. The main outcome for the task was the time it took for each participant to screw the nut onto the bolt using the left and right hand sequentially, both supported and unsupported. The independent variables were the three groups (Controls, PreHD, HD). Normality for all the dependent variables was tested using one-sample Kolmogorov-Smirnov tests. Group effects for variables that were normally distributed, such as age, CAG repeat, and UHDRS motor score were analysed using parametric tests (ANOVA) followed by post-hoc comparisons (T-test) with Bonferroni corrections where significance was present, all p values reported in the manuscript are already corrected. The group main effect was found to be significant. Variables with a skewed distribution, including data obtained from the nut and bolt assessments were first logarithmically transformed to obtain normality and analysed as above with the exception of the total functional capacity scores which were analysed using Mann-Whitney U tests. Partial Spearman correlations were performed using age as a covariate.

## RESULTS

**Participant demographics**

A total of 83 participants were tested on this task and were divided into 3 groups: healthy controls, preHD and manifest HD participants (See table 1). As would be expected, manifest HD participants had a higher UHDRS total score [p < 0.001] and a lower TFC score [p < 0.001] than preHD participants. Healthy controls and preHD participants displayed similar baseline demographics (age) [p = 0.863], while participants with manifest HD were slightly older than control participants [p = 0.004] (Table 1).

**Table 1:Demographic and clinical characteristics of study participants.** 

Group	Age	CAG	UHDRS	TFC
Premanifest (n=24)	47.47 ± 2.66	41.73 ± 0.662	3.87 ± 0.975	12.26 ± 0.492
Manifest (n=27)	56.30 ± 2.62*	43.74 ± 0.796	24.07 ± 2.28#	8.78 ± 2.28#
Controls (n=32)	43.08 ± 3.18	-	-	-

Table 1. Table shows mean ± S.E.M. UHDRS: Unified Huntington’s Disease Rating Scale: total motor score, TFC: total functional capacity. *indicates statistically significant difference when compared to controls whereas # indicates statistically significant difference when compared to preHD.PreHD participants show impaired performance in their performance with the non-dominant hand compared to controls.

**PreHD participants show impairments in their performance with the non-dominant hand compared to controls.**

PreHD participants were impaired on several aspects of the nut and bolt task when compared to the healthy controls, particularly when the non-dominant hand was being tested. Significant changes were observed with all sizes of the nut and bolt when the non-dominant hand was in the unsupported condition [small: p = 0.036. medium: p = 0.006 large: p = 0.001, table 2] and the large nut and bolt when the non-dominant hand was supported [p = 0.034]. In contrast, only one measurement was significantly different when testing the dominant hand [medium, unsupported: p = 0.009] indicating that dominant hand function is relatively well preserved at this stage of the condition (Table 2).

**Anti-dopaminergic medication has no effect on performance on the nut and bolt task.**

Anti-dopaminergic medications such as sulpiride and olanzapine are commonly used for treating the motor features of HD and hence may have an effect on performance in the nut and bolt task. In order to analyse the effect of such medications, we divided the preHD and HD participants into those who were receiving anti-dopaminergic medications and those who were not. Due to the nature of prescribing such medication for symptomatic relief of the motor features of HD, patients who were receiving such medication were in a more advanced stage of disease and hence were significantly older and a higher total motor score on the UHDRS [TMS: t = 3.29, p = 0.002, Age: t = 2.85, p = 0.006]. Therefore in order to minimise such confounding facts we included age and the total motor score as covariates in our analysis. Our results showed that there was no statistically significant difference in motor performance between those receiving anti-dopaminergic medication and those who were not [F = 1.75, p > 0.111].

**Table 2: Median times for each group to complete nut and bolt task; for all task conditions:** 

Median (Seconds)						p values		
			Controls	PreHD	HD	Control-PreHD	PreHD-HD	Controls-HD
Non Dominant	Supported	Small	24	29	37	0.435	0.024	<0.001
		Medium	19	25	31	0.076	0.04	<0.001
		Large	14.5	24	21	0.034	0.093	<0.001
	Unsupported	Small	19	25	36	0.036	0.032	<0.001
		Medium	14.5	23	30	0.006	0.198	<0.001
		Large	10	19	23.5	0.001	0.235	<0.001
Dominant	Supported	Small	23	25	43.5	0.152	0.002	<0.001
		Medium	19.6	23	35.5	0.122	0.021	<0.001
		Large	17.5	20	29	0.559	0.084	<0.01
	Unsupported	Small	21	22	36.5	0.239	0.02	<0.001
		Medium	16.5	21	32	0.009	0.052	<0.001
		Large	13	15	26.5	0.14	0.006	<0.001

**Manifest HD patients are significantly impaired in all domains of the nut and bolt task**

Compared to controls the manifest group performed worse on all conditions tested while the preHD group appeared to demonstrate deficits under specific conditions (Table 2). In particular, whereas dominant hand function was not significantly different when comparing preHD to controls, manifest HD participants showed impairments compared to preHD when the dominant hand was tested with the small and medium nut and bolts whilst supported [small: p = 0.002, medium: p = 0.021] and the small and large nut and bolts whilst unsupported [small: p = 0.02, large: p = 0.006]. Performance with the small nut and bolt using the unsupported non dominant hand is significantly impaired at all stages of disease [preHD compared to controls: p = 0.036, HD compared to preHD: p = 0.032] (Figure 1) and correlates significantly with disease burden score (r=0.374), even when controlled for age [p = 0.021] (Figure 2). Finally all components of the nut and bolt task correlated with the patients UHDRS score in both preHD and manifest patients [small non-dominant unsupported nut and bolt: r = 0.494, p = 0.001].

**Non dominant unsupported hand measurements of time to complete small nut and bolt test for each group.**
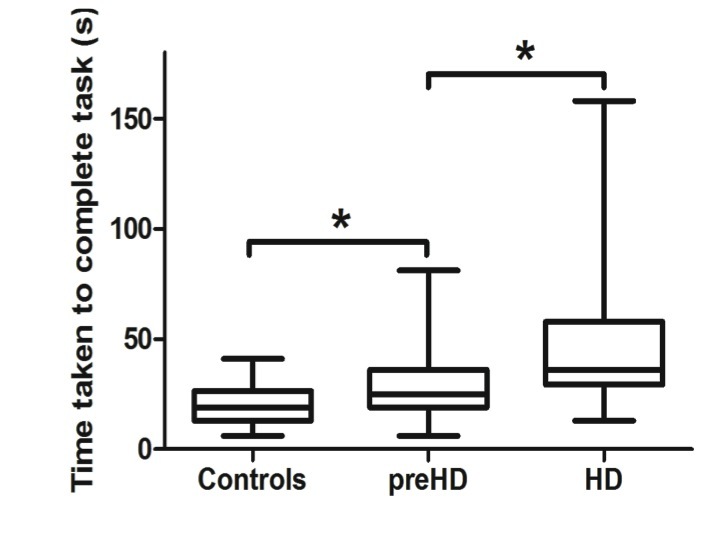
Unsupported small nut and bolt in the non dominant hand in each group. Significance is denoted by asterisks, preHD compared to controls: p = 0.036, HD compared to preHD: p = 0.032.

**Small nut and bolt measurement correlates with Disease Burden Score.**
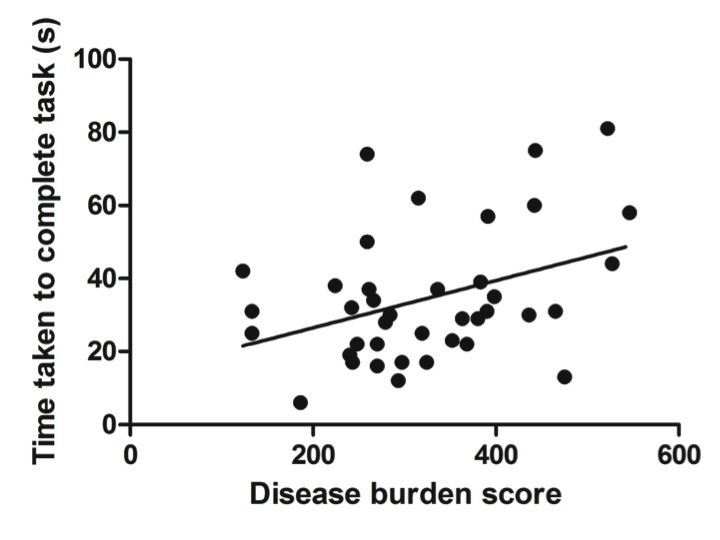
The time taken for the non dominant unsupported hand to complete the small nut and bolt task correlates with disease burden score [r = 0.374, p = 0.021]. Correlation is significant at the 0.05 level (2-tailed).

## Discussion

The ability to detect, quantify and follow the loss of hand dexterity in HD is of importance for diagnosing patients at the earliest stages of disease, monitoring disease progression and evaluating the efficacy of novel treatments. Previous tests that have been proposed to be useful in this regard include finger 8 ^,^ 9 and hand tapping 10 ^,^ 11 ^,^ 12 ^,^ 13 with both tasks tracking disease course over time 10 ^,^ 27 ^,^ 28. However, while they capture changes in motor speed they do not measure changes in dexterity. In our new study, we introduced a novel task: the nut and bolt test, which we have now shown to be a useful way of measuring the impact of HD on fine motor coordination in a large group of patients. In particular, we have found significant differences in performance between preHD and controls in their non dominant hand with the dominant hand function remaining intact until later in the disease course. Furthermore the non dominant nut and bolt performance correlated with disease burden scores suggesting a relationship with proximity to disease onset. Therefore, the nut and bolt task may be both diagnostically useful and helpful for identifying premanifest patients at immediate risk of developing manifest disease who may also therefore be suitable for future trials of disease modifying therapy.

This finding of decreased performance in the non dominant hand is in agreement with the existing literature; specifically the TRACK-HD study that showed that there is a reduction in finger tapping in the non dominant hand over time 28. In the cohort of premanifest and early HD patients, it was found that tapping was one of the few functional measurements that had significant differences in preHD participants compared to controls and also progressed over time 28. Previously, differences in performance on simple speed tapping tasks in preHD and early stage HD cohorts have been correlated with cortical thickness, disease burden scores and motor scores 18 and in another study manifest and early HD patients tapping scores correlated with cognitive test scores, regional brain atrophy and UHDRS scores 19. All of which highlights that these simple motor measures are useful measures of disease stage with a pathology that can also be quantified.

Other motor tasks that have also be used in a similar way in HD include saccadic eye movements in which it has been shown that preHD patients can have a number of abnormalities that seem to track disease course over time 17. Quantitative measurements of tongue protrusion force in preHD and HD have also detected deficits in motor force 15 and in motor speech timing tasks in manifest HD patients showing impaired speech rhythm with defects in speech rate, increased pauses, impaired syllable repetition all of which correlated with motor tapping assessments 16. Other studies have shown slowing of rapid alternating movements in HD patients, as well as deficits in tapping and on peg insertion tasks 20. This confirms earlier findings where the peg insertion task was significantly different in HD patients compared to controls but failed to detect early changes in preHD groups 21. These tasks along with our simple task may suggest that the combination of speed and simple movements may highlight early deficits in HD, especially in the less dextrous non dominant hand. Indeed our nut and bolt task is unique in that is takes into account speed, dexterity, hand movements and motivation and as such may be more sensitive to the very earliest problems in HD which effects all of these functions. This would fit with imaging and post mortem data showing that those brain regions involved in these motor and affective activities are known to be abnormal in preHD and early HD including the striatum 28 ^,^ 29 ^,^ 30 the nucleus accumbens, pallidum 31 precentral and postcentral gyri as well as the supplementary motor area 32. Thus the clinical data sits well with the known neuropathology of premanifest and early HD.

Although, our test has many advantages including its cost and ease of administration and simple interpretation of results there are also some limitations with our study . This includes the absence of longitudinal data, and the need to trial it in larger numbers of patients and the problems of employing it in very advanced patients. However, this latter group are unlikely to be the target of new therapeutic interventions in the first instance, so this is less of an issue. Furthermore the nut and bolt task was timed manually using a standard stop watch and although the timing was not automated, each individual was trained in the same way and thus is unlikely to have contributed to any significant issues on the accuracy of the data collection. However using an automated timer may be a useful addition to our task. Finally our study captured a selection of un-medicated and medicated preHD and HD patients seen in our regional clinic, including HD patients on anti-dopaminergic medication such as sulpiride and olanzapine. Although we found no significant difference in our task in those patients on anti-dopaminergic medications compared to those who were not, we cannot rule out that this may effect their performance. In the future longitudinal studies that specifically address this issue are needed.

In summary, we describe a simple, inexpensive and robust task that is useful in defining disease onset as well as being of possible value in therapeutic trials of disease modifying therapies in both manifest patients and pre-HD patients approaching the time of phenoconversion. Furthermore it can be used in any clinic on its own or as part of a battery of tests to assess dexterity across all stages of HD given its simplicity and low cost.

## Competing Interests

The authors have declared that no competing interests exist.

## Author Information

Lucy M. Collins and Faye Begeti contributed equally to this work.
